# Walk or be walked by the dog? The attachment role

**DOI:** 10.1186/s12889-024-18037-4

**Published:** 2024-03-04

**Authors:** Catarina F. Martins, Luís Silva, Jorge Soares, Graça S. Pinto, Catarina Abrantes, Luís Cardoso, Maria A. Pires, Hélder Sousa, Maria P. Mota

**Affiliations:** 1 Research Centre in Sports Sciences, Health, and Human Development (CIDESD), University of Trás-os- Montes and Alto Douro (UTAD), Vila Real, Portugal; 2 Department of Sport, Exercise and Health Sciences, School of Life and Environmental Sciences (ECVA), UTAD, Vila Real, Portugal; 3 Animal and Veterinary Research Centre (CECAV), UTAD, and Associate Laboratory for Animal and Veterinary Sciences (AL4AnimalS), Vila Real, Portugal; 4 Department of Veterinary Sciences, School of Agrarian and Veterinary Sciences (ECAV), UTAD, Vila Real, Portugal; 5 Department of Mathematics (DM), UTAD, Vila Real, Portugal; 6 Center for Computational and Stochastic Mathematics (CEMAT), Lisbon, Portugal

**Keywords:** Accelerometry, Companion animal, Dogs, Health, Lifestyle, Pet ownership, Physical activity

## Abstract

**Background:**

The human-animal bond has been recognized as having positive effects on the health and well-being of both humans and pets. The present study aims to explore the influence of attachment on physical activity (PA), lifestyle, and health outcomes of dog owners (DO), highlighting the mutual benefits resulting from the relationship between DO and dogs.

**Methods:**

Thirty-eight DO and their dogs participated in this study. Socio-demographic data, the Self-Rated Health (SRH), FANTASTICO Lifestyle Scale, and the Lexington Attachment Pet Scale (LAPS) were assessed. PA was measured in both the DO and the dogs, using an ActiGraph GT3X accelerometer in the context of daily routine. Descriptive statistics and Spearman rank correlation analyses were performed to examine the associations between LAPS, PA levels, socio-demographic variables, lifestyle behaviors, and SRH.

**Results:**

Significant correlations were found between the dog owners’ light-level PA and the pets’ vigorous level of PA (rho = 0.445, *p =* 0.01). Furthermore, the importance of the pets’ health (rho = -0.785, *p* = 0.02) and the LAPS subscales, namely proximity (rho = 0.358, *p* = 0.03), and attachment (rho = 0.392, *p* = 0.01), were related to taking the pet for a walk. Regarding lifestyle, DO with a healthier lifestyle had a better self-assessment of their health using the SRH (rho = 0.39, *p* = 0.02). Moreover, DO with better lifestyles also exhibited greater concern for their pet’s health (rho = 0.398, *p* = 0.01).

**Conclusions:**

This study emphasizes that individuals who adopt healthier habits tend to perceive themselves as healthier and exhibit greater concern for their pets’ health. The attachment between DO and dogs is important in promoting healthy lifestyle behaviors and engagement in PA. Our results highlight that the presence of a dog is associated with a higher level of PA in DO, depending on the strength of the human-animal bond.

**Supplementary Information:**

The online version contains supplementary material available at 10.1186/s12889-024-18037-4.

## Introduction

The comprehensive implementation of health prevention and health promotion policies, raising awareness of modifiable risk factors, and promoting healthy lifestyles represent an ever-evolving challenge for today’s society [1, 2]. Lifestyle plays a crucial role in public health, as individual behaviors and habits have a significant impact on the development of chronic non-communicable diseases [1, 3]. Adopting a healthy lifestyle, including a balanced diet, regular physical activity (PA), abstaining from tobacco, moderate alcohol consumption, and getting restful sleep, can help prevent and control these diseases, reducing the burden on health systems and improving the quality of life of the population [4, 5].

Exercise levels in the global population are a public health concern and are considered one of the greatest challenges for the future [1]. An analysis conducted in 2018 found that 27.5% of adults worldwide were insufficiently active [6]. The latest Eurobarometer data indicate that 73% of Portuguese people report that they never exercise or engage in PA [7]. Several strategies have been implemented to increase the level of PA and reduce sedentary behavior, such as the creation of community-based walking groups, public policies [8], and including a pet during PA [9].

In 2010, the World Small Animal Veterinary Association requested that the One Health Agenda consider the effects of pets on humans [10]. In a One Health approach, the human-animal bond plays a prominent role. The concept of the human-animal bond refers to the positive effects that result from the relationship between an owner and their pet, where each one influences the psychological, physical, and physiological state of the other [11]. Several benefits associated with PA have been described for both owners and their pets [12–15]. Pets are present in almost half (54%) of Portuguese households [16]. The use of pets, particularly dogs, as a source of encouragement and motivation for PA has received increasing scientific attention [14, 17–21], and has been proposed as a strategy for promoting PA in the population [17].

Several studies have reported that dog owners (DO) engage in more total walking compared to non-dog owners [9, 18, 22, 23]. In a meta-analysis conducted by Martins et al. (2023), pet ownership was found to have a moderately significant positive effect on the PA of pet owners compared to non-pet owners [24]. However, while increasing evidence indicates that dog ownership influences increased PA, other studies find that many owners do not take their dogs for walks. In 2018, Christian et al. [17]reviewed the evidence from longitudinal and experimental studies and found only two changes in PA with the acquisition of a dog. This inconsistency found between these studies may rely on methodological issues such as the use of self-reported instruments, which may be susceptible to bias [18, 21, 24, 25, 26, 27, 28].

Martins et al. (2023) have identified several variables that can influence the health benefits of owning a pet [24]. One possible factor is the level of attachment to the pet [29]. It has long been hypothesized that the bond between pet and owners, particularly DO, plays a crucial role in the relationship between pet ownership and human health [30, 31]. Furthermore, it is recognized that most DOs believe that regularly walking their dogs is beneficial for the human-animal relationship and for the dog’s health [32]. Walking a dog helps individuals experience fewer negative emotions and promotes emotional stability through social and emotional support [33]. This human-animal relationship may resemble the role that human social support plays in facilitating the initiation and maintenance of PA. Additionally, the owner’s overall caregiving strategy, when evaluated, could play a role in understanding the relationship developed between the owner and the dog [34].

Given the substantial prevalence of dog ownership within Portuguese households and the prevailing animal welfare initiative aimed at rehoming adoptable shelter animals, there are numerous opportunities to harness the human-canine bond in order to promote elevated levels of PA.

With the growing development of technological innovations, numerous user-friendly devices provide the opportunity to assess PA levels [35]. Accelerometers have been used to quantify PA and estimate energy expenditure in both owners and pets [36–39]. Furthermore, the use of accelerometers has been validated as an objective and representative measure of spontaneous activity in humans and dogs [38, 40–44].

Potential benefits of accelerometry data, when compared to traditional pet activity monitoring, include excellent owner and dog compliance, as well as the ability to obtain objective and unbiased data from owner and researcher observations [42]. Indeed, the reliance on subjective measures of PA in studies is a concern. In a meta-analysis conducted in 2013 [18], only 4 out of 17 studies considered the use of an objective measure of PA. This concern was further discussed in a more recent meta-analysis [24],where it was found that only 8 out of 27 studies included more objective measures of PA. These findings highlight the importance of conducting studies that provide more objective measurements of PA. Additionally, accelerometry allows for the most realistic monitoring of daily PA [36]. Although PA monitoring in dogs is often performed in controlled environments [45], assessing PA in daily-life contexts enables closer monitoring of real-life situations, as the types of activities that dogs engage in likely vary substantially between households [46].

Various sociodemographic aspects, such as the size of the dog, the gender of both the owner and the dog, and the number of people in the household, among other factors, can influence daily PA for both dogs and humans [23, 47]. Owners with a higher level of education seem to achieve better health outcomes and tend to take better care of animal welfare [48]. Including these factors can significantly shape our understanding of the relationship between owning a dog and engaging in PA [49]. This is particularly relevant in Portugal, where, to our knowledge, no studies have explored the relationship between these variables and the practice of joint PA between owners and dogs. In addition, dog ownership has also been empirically linked to positive mental health outcomes [50], social benefits [51], and positive health behaviors [52].

This study was conducted in the daily life context of DO and their dogs to explore the unique relationships between both influences. The purpose of this research was to examine whether the attachment between DO and their dogs influenced total PA, as measured by accelerometry. Additionally, we examined the relationship between socio-demographic variables, lifestyle, the SRH of the DO and the health care of their dogs.

## Methods

### Ethics statement

This study has received approval from the UTAD Ethics Committee for the project “One health approach in animal cancer Norte-01-0145-FEDER-000078” (Ref.: Doc55-CE-UTAD-2021), and it adhered to the principles outlined in the Declaration of Helsinki. All DO who participated in the study provided written informed consent and were informed of their right to voluntarily withdraw from the study at any time.

### Participant recruiting

During the first phase, the sample was obtained through convenience and snowball sampling. Participants were contacted via social networks, and approached DO while they were walking in public streets or parks. Additionally, participants were recruited though the word of mouth.

After recruiting potential participants, an initial face-to-face meeting was arranged to explain all stages and procedures of the study. This meeting also ensured that all participants met the requirements for inclusion in the study.

The inclusion criteria were owning a dog as a pet, and both the DO and the dogs residing in the same household. The exclusion criteria included the absence of chronic musculoskeletal pain or any other health condition that could interfere with daily PA, particularly walking, for both dogs and owners. Non-compliance with intervention procedures, such as not using the accelerometer for five consecutive days for both the DO and the dog, as well as not completing the questionnaires, was also a factor. Additionally, DO who had no daily contact with their dog or left the dog unsupervised were excluded to protect the equipment.

In addition, a protocol was implemented to ensure that most of the researchers were unaware of the participants’ identities. Only one team member had direct contact with the participants, while the other members, involved in various other tasks such as data acquisition, had no access to information that could reveal the participants’ identities.

**Fig. 1:**
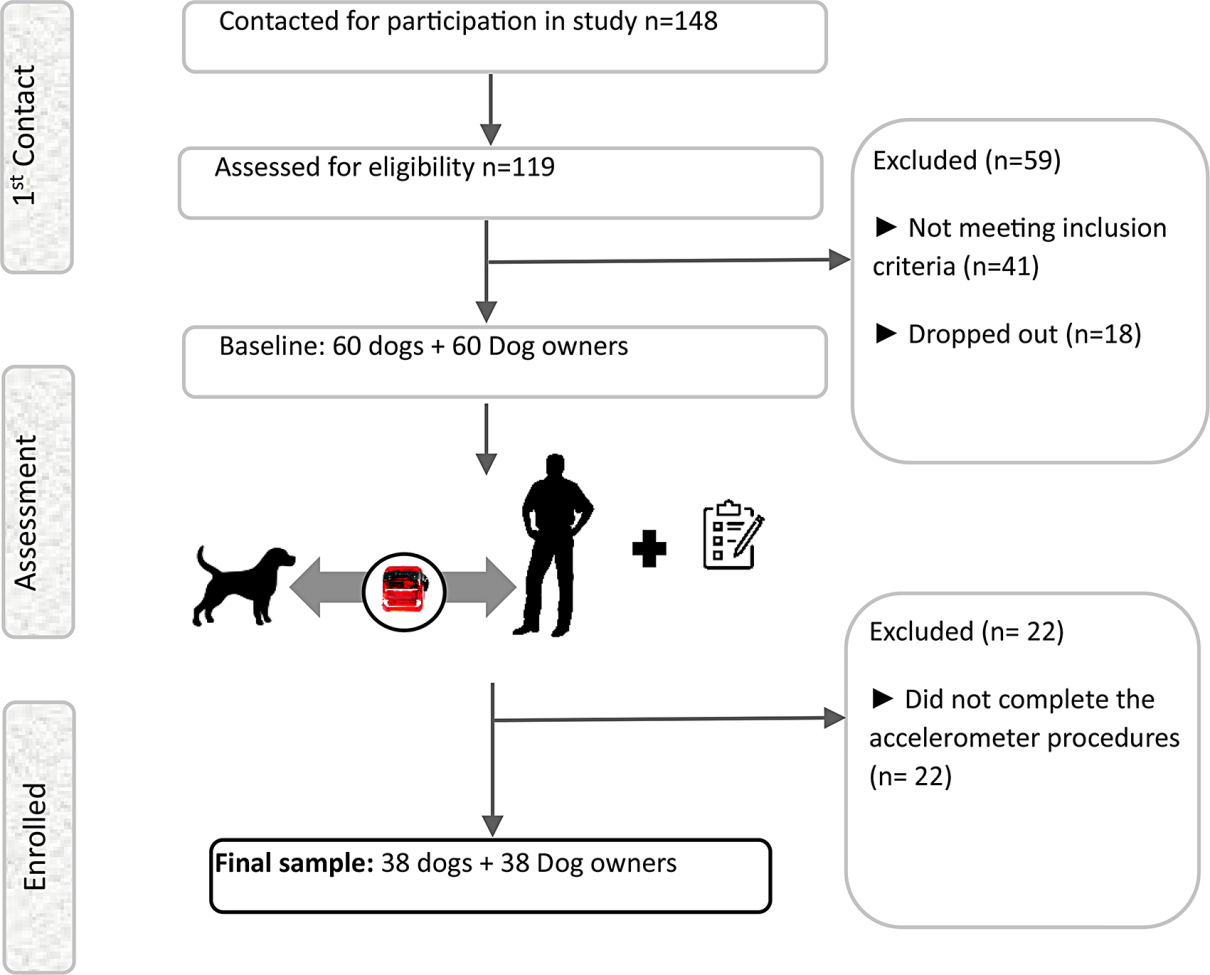
Flowchart of the study sample

The initial sample for this study comprised 148 adults. However, only 60 participants proceeded with the intervention (Fig. 1).

### Procedure

In the second phase of the study, each participant (including both dogs and owners) was assigned a unique numeric identification code. After completing an online questionnaire, participants were invited to move on to the third phase of the study. This phase aimed to measure the PA levels of both the owners and their dogs using accelerometry.

Before starting this phase, a face-to-face meeting was scheduled with each participant and their pet to introduce the use of accelerometers. Each participant received a kit that contained the accelerometers, marked with their unique identification codes. The kit also included written instructions explaining how to use the accelerometers, as well as a daily log sheet for recording accelerometer usage.

Once all participants were adequately prepared and had been instructed on how to use the accelerometers, they began using them and documented their PA on the daily log sheet. Throughout this period, regular contact was maintained with the participants. After the conclusion of this phase, all equipment was collected.

Following the intervention, 22 participants were excluded from the study due to incomplete accelerometer procedures.

### Measures

#### Socio-demographic and health information

##### Dog owners

The socio-demographic features of the DO, including age, sex, and number of household members, were collected through a self-reported demographic questionnaire. Participants reported their educational level by selecting from three categories: basic school, high school degree, bachelor’s degree or higher. Regarding the occupations/employment the participants were grouped by economic sectors [72, 73]. The primary sector refers to jobs related to natural resources, the secondary sector refers to jobs related to manufacturing and construction, and the tertiary sector refers to jobs related to services. The DO’s body mass index (BMI) was calculated using their self-reported weight and height [53]. BMI z-scores were determined using reference data provided by the World Health Organization (WHO) [54].

Participants’ self-rated health (SRH) was assessed using two items to measure general health. The questions asked were “How is your current health status?” and “How do you rate your health compared to others your age?”. Response options for both questions ranged from 1 (Very poor) to 5 (Excellent) [47]. In the current sample, the self-rated health (SRH) showed high internal consistency, as reflected in Cronbach’s alpha (α) = 0.89.

##### Dogs

The socio-demographic characteristics of the canine participants, including age, sex, time of adoption, and dog care practices (such as deworming, sterilization, and vaccination), were collected. Additionally, DO were asked about the importance of their dog’s health, with response options ranging from 1 (Not important at all) to 5 (Very important). The researcher assessed the dog’s body condition index using a semi-quantitative subjective method during the contact with both the participants and their dogs. This method takes into account visual characteristics from both the top and side views, as well as palpable characteristics such as the ribcage, dorsal spinous processes, and waist. These factors are used to estimate the degree of obesity [55–57]. The Body Condition Score (BCS) is typically rated on a scale from 1 to 9, with 1 indicating very thin and 9 indicating very obese [56, 58]. The ideal body condition index for a dog is typically between 4 and 5. This means that the dog should have a visible waist when viewed from above, ribs that can be felt but not seen, and a slightly rounded abdomen [56, 57]. The weight range for each breed was considered and grouped into three size categories: small (less than 10 kg), medium (10 kg to less than 25 kg), and large (25 kg or more). These categories were based on widely applied criteria [59, 60].

#### Physical activity

##### Dog owners

PA levels for both the DO and dogs were measured by accelerometry in their home or routine environments using an ActiGraph wGT3X-Link accelerometer [61]. The ActiGraph accelerometer is a small, lightweight device that detects motion and records information on the intensity, frequency, and duration of movement, as well as sedentary time [61].

In this study, the ActiGraphs were set to a sampling rate of 30 Hz [61] and placed on the non-dominant wrist of the DO. Despite being used 24 h daily, the presented data will reflect on daytime wear period, from 6:00 AM to 11:00 PM [62]. After data collection, the devices were downloaded, processed, and analyzed using ActiLife v6.13.4 software (ActiGraph Inc., Pensacola, FL, USA). Mean values for each intensity level of PA were calculated for free-living adults using the cut-points proposed by Montoye et al. [38]: sedentary (< 2.860 counts/min), light PA (LPA) (2.860–3.940 counts/min), and moderate-to-vigorous PA (MVPA) (≥ 3.941 counts/min).

All study participants were required to record the times they used and removed the accelerometer in a PA diary, both for themselves and their pets. To ensure the accuracy of the data, the ActiGraph data needed to show at least 10 h of wear time per day for a minimum of 5 days. The total amount of LPA, MPA and, MVPA, as well as the number of steps, recorded during the week, were divided by the number of valid wear days for each participant to calculate daily averages.

Additionally, we followed the PA recommendations of the WHO [63], which suggest an average of at least 150–300 min of MVPA per week.

##### Dogs

For the dogs, we used the canine protocol, where a ActiGraph accelerometer was attached to each dog’s harness or ventrally on the collar, following the methodology used in previous studies [36, 39, 42]. The accelerometers were set to record activity in 15-second intervals (epochs). Furthermore, the results of Westgarth and Ladha [64] provide strong evidence that both the ventral collar and dorsal harness sites can be used interchangeably, as there was low variability observed between the two locations [64].

The dog´s ActiGraph data was downloaded using the Low-Frequency Extension option [45] and then screened for wear time using the graphing function of ActiLife v6.13.3 software. Validated cut-points from Morrison et al. 2013 [12], were used to set different intensity levels: sedentary (< 1351 counts/min), moderate PA (MPA) (1352–5695 counts/min), and vigorous PA (VPA) (> 5696 counts /min) (12,39).

Canine participants’ PA was also assessed using owner-reported measures. These measures were based on the average time spent per day presented in ranges and included duration of walks (with response options such as “Do not walk the dog”, “Less than 15 minutes”, “Between 16 and 30 minutes” or “More than 30 minutes”) and the amount of time spent alone (with response options “Less than 3 hours”, “Between 3 and 6 hours” and “Between 6 and 12 hours”). The frequency (number of times the dog was taken out) was also collected, with options ranging from 0 (never) to 5 or more times per day.

#### Lifestyle

The FANTASTICO Scale [65] was used to assess lifestyle. The Portuguese version of the scale, which has been adapted and validated, consists of 30 questions divided into 10 subscales that cover various aspects of lifestyle. The subscales include Family and Friends, Associativism/Physical Activity, Nutrition, Tobacco, Alcohol and other drugs, Sleep/Stress, Type Personality/Work, Introspection, Health and Sexual Behaviors, and Other Behaviors. The questions were designed to be answered on a rating with numeric values of 0, 1, or 2 [66].

The raw score obtained from the questionnaire was transformed into a scale ranging from 0 to 120 points, which allows for behavior to the different categories. Scores from 0 to 46 indicate a need for improvement, scores from 47 to 72 suggest a fair lifestyle, scores from 73 to 84 represent a good lifestyle, scores from 85 to 102 indicate a very good lifestyle, and scores from 103 to 120 reflect an excellent lifestyle. A lower score suggests a greater need for behavior change.

The interpretation of the results is as follows: an “Excellent” score means that the lifestyle has a significant positive influence on health. A “Very good” score suggests that the lifestyle will have an adequate influence on health; “Good” indicates that the lifestyle will bring numerous health benefits. A “Fair” score implies that the lifestyle provides some health benefits, but also presents certain risks, and a “Needs improvement” score indicates that the lifestyle has several risk factors [66].

In the current sample, the internal consistency of the FANTASTICO Scale was high, as reflected in Cronbach’s alpha (α) = 0.81.

### Attachment

The Lexington Attachment to Pets Scale (LAPS) was developed by Johnson et al. in 1992, using Social Support Theory as the underlying theoretical framework [67]. The scale was designed to capture the affective aspect of the pet’s supportive role in the owner’s life, as this aspect is considered to have a strong impact on human well-being and psychophysical health [68, 69]. The original questionnaire consists of 23 items, divided into three sub-scales: “General Attachment”, “People Replacing”, and “Animal Rights/Animal Welfare” [67]. Each item is scored from 0 (strongly disagree) to 3 (strongly agree) [67]. The Portuguese version, while also using 23 items, differs slightly. Each item is rated on a scale of 1 to 5, ranging from “Strongly disagree” to “Strongly agree” (Additional file 1). This version includes three subscales: Proximity, Importance, and Bond. Together these provide a comprehensive measure of attachment to pets [70]. The overall level of attachment is determined by summing the scores obtained for each item. In the current sample, the LAPS demonstrated high internal consistency, as reflected in Cronbach’s alpha (α) = 0.82.

### Statistical analyses

Data analysis was conducted using SPSS Statistics 27.0 software (IBM, Inc., Chicago, I.L, USA). Descriptive statistics (frequency and percentages for categorical variables, mean, and standard deviation for continuous variables or median, interquartile range (*IQR*), and minimum/maximum when continuous variables were not normally distributed and non-parametric statistics were used) were used to analyze socio-demographic characteristics, health-related characteristics, and accelerometer-measured PA in dogs and DO. The employment variable was then divided into two categories, employed and unemployed, to examine the association between this variable and owner-reported PA using the Fisher Exact Test. This dichotomization was also used to compare accelerometer-measured PA in dogs using the Mann-Whitney test, given the non-normal distribution of the accelerometer-measured PA data. To explore disparities in canine PA levels based on education levels, the Fisher Exact Test was used for self-reported PA, and the Kruskal-Wallis test was used for objectively PA, again due to the non-normal distribution of the accelerometer-measured PA data.

Spearman rank correlation was employed to assess the relationship between variables such as SRH, PA, LAPS, and lifestyle. This method was chosen because all variables were either ordinal (SRH, LAPS, lifestyle) or non-normally distributed continuous data (accelerometer-measured PA). The criteria proposed by Cohen (1988, 1992) were used to interpret the magnitude of the correlation measures, with recommended Pearson *r* values of 0.10, 0.30, and 0.50 indicating small, medium, and large effects, respectively [71]. Statistical significance was considered at *p* ≤ 0.05. Due to the exploratory nature of the study, a multivariate approach was not supported by the data.

## Results

### Participants of study

#### Dog owners

The mean age of the participants was 43.1 ± 16.6 years, with 65.8% of the sample being female. In terms of household composition, twenty-seven individuals lived with three or more people, seven reported living with their partners, and four reported living alone. When considering Body Mass Index (BMI) (kg/m2), 60.5% of our participants had a normal weight, 26.3% were pre-obese, and 13.5% were obese. Regards to of educational level, 60.5% of the participants had a bachelor’s degree or higher, followed by 21.1% who completed high school. The remaining participants (18.5%) frequented the basic school level. Concerning the participants’ occupation/employment, 70.1% worked in the tertiary sector, 7.9% in the secondary sector, while the remaining individuals were students (15.8%) or retired (5.3%). None of the participants reported any chronic illnesses that would hinder their participation in PA. The results regarding lifestyle, pet attachment, self-perceived health, and accelerometry data can be found in Table 1.

**Table 1:** Mean, median, interquartile range (IQR), minimum/maximum scores and baseline characteristics of dog owners (DO) participating in the study

Variables		DO (*N* = 38)		
		Mean (SD)	Median (IQR)	Min/Max
**FANTASTICO**	F -Family and Friends	7.05 (± 0.24)	8.00 (6.00–8.00)	4.00–8.00
	A -Associativism/Physical Activity	7.95 (± 0.45)	8.00 (6.00-10.50)	4.00–12.00
	N -Nutrition	7.52 (± 0.34)	8.00 (6.00-8.50)	2.00–10.00
	T -Tobacco	6.73 (± 0.36)	8.00 (5.50-8.00)	2.00–8.00
	A -Alcohol and other drugs	21.37 (± 0.52)	22.00 (20.00–24.00)	14.00–24.00
	S -Sleep/Stress	9.11 (± 0.40)	10.00 (7.50–12.00)	2.00–12.00
	T -Type Personality/Work	7.16 (± 0.39)	6.00 (6.00–10.00)	4.00–12.00
	I -Introspection	7.63 (± 0.49)	8.00 (4.00–10.00)	2.00–12.00
	C -Health and Sexual Behaviors	9.31 (± 0.30)	10.00 (8.00–10.00)	6.00–12.00
	O -Other Behaviors	7.00 (± 0.26)	8.00 (6.00–8.00)	4.00–8.00
	Total Scale	90.84 (± 2.15)	92.00 (79.50–102.00)	62.00-114.00
**LAPS**	Proximity	25.74 (± 4.86)	27.50 (22.75-29.00)	13.00–31.00
	Importance	20.21 (± 3.59)	20.50 (17.00-23.25)	14.00–25.00
	Bond	48.87 (± 3.81)	50.00 (46.00–51.00)	39.00–55.00
	Total Scale	94.82 (± 10.88)	97. 00 (88.50-103.25)	72.00-107.00
**Accelerometer data** ^1^	METs (kcal/kg/h)	1.83 (± 0.35)	1.72 (1.58–1.72)	1.38–2.71
	Total sedentary time (min/day)	1005.71 (± 101.03)	1011.81 (9.31.63-1081.45)	771.27-1200.83
	LPA (min/day)	89.83 (± 53.93)	80.43 (69.74–85.06)	39.40-377.20
	MVPA (min/day)	246.71 (± 94.28)	232.30 (178.84-311.66)	17.40-490.21
	Total steps count (counts/day)	12,469 (± 3708)	11954.00 (9253.25-15540.75)	7587–21,370
**N (%)**				
**SRH** Q1 “Current health”	Average Good	1 (2.6%) 25 (65.8%)	- -	- -
	Excellent	12 (31.6%)	-	-
	Excellent	12 (31.6%)	-	-
Q2: “Health compared to others”	Average Good Excellent	1 (2.6%) 25 (65.8%) 12 (31.6%)	- - -	- - -

Abbreviations kcal/kg/h = Kilocalorie per kilogram per hour, METs = Number of metabolic equivalents, min/day = Minute per day, counts/day = counts per day, LPA = Light PA, MVPA = Moderate-to-vigorous PA. *SD* = Standard derivation.^1^ Calculate by cut points Montoye et al., 2020.

#### Dogs

The 38 dogs under study presented a wide age range: 13.2% were 2 or less than 2 years old, 65.8% were between 3 and 8 years old, and 21.1% were 9 or more years old. Regarding breed, 30 dogs were of no defined breed, while the remaining 8 dogs belonged to specific breeds (2 Jack Russell Terriers, 1 Afghan Hound, 1 Cavalier King Charles Spaniel, 1 Yorkshire Terrier, 1 Schnauzer, and 2 Miniature Pinschers). These dogs were further classified into 13 small dogs, 19 medium dogs, and 6 large dogs. None of the dogs included in the study had any chronic diseases that would prevent them from participating in PA.

Based on BCS, 23.7% of the dogs were underweight, 55.3% were overweight, and 21.1% had an optimal weight. In terms of feeding frequency, 10.5% of DO feed their dogs once a day, 47.4% fed them twice a day, 21.1% fed them three times a day, and 21.1% kept food available for their dogs at all times. Additionally, 76.3% of DO had neutered their dogs, and 86.8% made sure their dogs were up-to-date with vaccinations. All DO-reported deworming their dogs for internal and external parasite. The length of time since the dogs were adopted ranged from 1 to 18 years, with an average of 5.58 ± 3.4 years. When asked about the importance of their pet’s health, all DO indicated that it was either ‘Important’ or ‘Very important’. The owner-reported information about the PA of their dogs and the results from accelerometry are provided in Table 2.

**Table 2:** Mean, median, interquartile range (*IQR*), minimum/maximum scores and owner-reported information of dogs’ physical activity (PA) variables

Variables		DOGS (*N* = 38)		
		Mean (SD)	Median (IQR)	Min/Max
**Accelerometer data** ^**1**^	METs (kcal/kg/h)	1.05 (± 0.04)	1.04 (1.02–1.08)	1.00-1.16
	Total sedentary time (min/day)	1233.95 (± 87.01)	1256.20 (1140.63-1303.73)	1060.17-1380.60
	MPA (min/day)	86.34 (± 59.54)	74.74 (40.70-111.23)	8.53-251.17
	MVPA (min/day)	89.49 (± 61.47)	78.45 (41.37-111.85)	9.13-259.83
	VPA (min/day)	3.14 (± 2.91)	1.83 (0.67–6.17)	0.03–8.67
	Total steps counts (counts/day)	9128 (± 4015)	9830.00 (6353.00-11690.05)	1154–21,325
**N (%)**				
**Owner-reported information**				
**Time dogs spend alone (h/day range)**	Less than 3 h	7 (18.4%)	-	-
	Between 3 and 6 h	21 (55.3%)	-	-
	Between 6 and 12 h	10 (26.3%)	-	-
**Frequency of walks** **(nº/day range)**	Never	1 (2.6%)	-	-
	1 time	2 (5.3%)	-	-
	2 times	9 (23.7%)	-	-
	3 times	12 (31.6%)	-	-
	4 times	3 (7.9%)	-	-
	5 or more times	11 (28.9%)	-	-
**Duration walking (min/day range)**	Does not walk the pet Less than 15 min	1 (2.6%) 6 (15.8%)	- -	- -
	Between 16 and 30 min	27 (71.1%)	-	-
	More than 30 min	4 (10.5%)	-	-

Abbreviations kcal/kg/h = Kilocalorie per kilogram per hour, METs = Number of metabolic equivalents, min/day = Minute per day, counts/day = counts per day, nº/day = Number per day, MPA = Moderate PA, MVPA = Moderate-to-vigorous PA, VPA = Vigorous PA. *SD* = Standard derivation. ^1^ Calculate by cut points Morrison et al., 2013.

Taking into consideration the measurements of PA for both the owners and their dogs throughout a 24-hour day, an example that demonstrates potential trends in simultaneous PA is presented in the additional file 2.

### Inferential analysis of study variables

#### Socio-demographic and physical activity

Regarding the sociodemographic variables employment status and education level of the owners when analyzed by the dogs’ PA levels measured by accelerometer (Table 3), it was found that dogs belonging to the employed owners’ group had significantly higher daily METs (U = 53.00; *p* = 0.015), daily step counts (U = 58.00; *p* = 0.026) and levels of MPA (U = 48.00, *p* = 0.08) and MVPA (U = 48.00; *p* = 0.08) compared with dogs of the unemployed owners group.

**Table 3:** Mean, median, interquartile range (IQR), minimum/maximum scores of dog accelerometer data by owner employment status and education level

Accelerometer-Measured PA of Dogs	Employment Status				Education Level					
	Unemployed		Employed		Basic School		High School Degree		Bachelor’s Degree or Higher	
	Median (IQR)	Min/Max	Median (IQR)	Min/Max	Median (IQR)	Min/Max	Median (IQR)	Min/Max	Median (IQR)	Min/Max
**METS (kcal/kg/h)**	1.02 (1.01–1.03)	1.00-1.11	1.05 (1.02–1.09)	1.01–1.16	1.02 (1.01–1.02)	1.00-1.05	1.03 (1.02–1.08)	1.02–1.11	1.05 (1.03–1.09)	1.01–1.16
**Total Sedentary Time (min/day)**	1209.48 (1134.63-1324.63)	1081.80-1334.87	1256.20 (1183.28-1297.10)	1060.17-1380.60	1264.37 (1134.63-1334.87)	1134.63-1380.60	12.29.76 (1138.08-1295.42)	1081.80-1308.30	1260.40 (1194.90-1295.40)	1060.17-1347.20
**MPA (min/day)**	40.70 (19.23–51.35)	8.53-229.93	85.90 (63.40-113.11)	18.93-251.17	40.70 (18.93–101.90)	8.53–113.10	81.00 (59.50-167.73)	40.70-229.93	76.83 (46.97-113.13)	18.93-251.17
**VPA (min/day)**	0.67 (0.028–3.75)	0.03–8.27	2.07 (0.94–6.45)	0.17–8.67	0.67 (0.60-3.00)	0.17–3.77	2.78 (0.71–5.74)	0.45–8.27	1.83 (0.67–6.90)	0.03–8.67
**MVPA (min/day)**	41.37 (19.37–55.09)	9.13–238.20	92.40 (64.28-114.98)	19.10-259.83	41.37 (19.10-103.63)	9.13–116.10	83.12 (61.23-173.47)	41.37–238.20	83.60 (48.60-114.97)	9.10-259.83
**Total Steps (counts/day)**	7200.60 (2607.40-7667.25)	1154.00-10564.00	10311.80 (6602.00-12146.95)	1430.00-21325.00	7200.60 (1430.00-10366.20)	1154.00-12311.00	10007.20 (6813.90-11955.15)	6189–14160.00	10257.40 (6353.00-11995.00)	1430.00-21325.00

Abbreviations kcal/kg/h = Kilocalorie per kilogram per hour, METs = Number of metabolic equivalents, min/day = Minute per day, counts/day = counts per day, MPA = Moderate PA, VPA = Vigorous PA, MVPA = Moderate-to-vigorous PA.

The METs of the dogs showed statistically significant variation depending on the owners’ level of education (H (2) = 7.940, *p* = 0.019). However, no statistical significant differences were observed in the other parameters.

When analyzing the same sociodemographic variables of owners according to owner-reported dog PA (Table 4), it was found that, in terms of the frequency of walks with dogs, employed owners were positively associated with walking their dogs one to three times per day. Conversely, unemployed owners were positively associated with walking their dogs four or more times per day.

**Table 4:** Owner-reported information of dogs’ physical activity (PA) by owner employment status and education level

Owner-Reported PA of Dogs		Employment Status		Education Level		
		Unemployed n (%)	Employed n (%)	Basic School n (%)	High School Degree n (%)	Bachelor’s Degree or Higher n (%)
**Time dogs spend alone (h/day range)**	Less than 3 h	2 (5.3%)	5 (13.2%)	3 (7.9%)	1 (2.6%)	7 (7.9%)
	Between 3 and 6 h	3 (7.9%)	18 (47.4%)	2 (5.3%)	4 (10.5%)	15 (39.5%)
	Between 6 and 12 h	3 (7.9%)	7 (18.4%)	2 (5.3%)	3 (7.9%)	5 (13.2%)
**Frequency of walks (nº/day range)**	Never	1 (2.6%)	0 (0.0%)	1 (2.6%)	0 (0.0%)	0 (0.0%)
	1 time	1 (2.6%)	1 (2.6%)	1 (2.6%)	0 (0.0%)	1 (2.6%)
	2 times	0 (0.0%)	9 (23.7%)	3 (7.9%)	2 (5.3%)	4 (10.5%)
	3 times	1 (2.6%)	11 (28.9%)	0 (0.0%)	2 (5.3%)	10 (26.3%)
	4 times	1 (2.6%)	2 (5.3%)	0 (0.0%)	0 (0.0%)	3 (7.9%)
	5 or more times	4 (10.5%)	7 (18.4%)	2 (5.3%)	4 (10.5%)	5 (13.2%)
**Duration walking (min/day range)**	Does not walk the pet	1 (2.6%)	0 (0.0%)	1 (2.6%)	0 (0.0%)	0 (0.0%)
	Less than 15 min	2 (5.3%)	4 (10.5%)	2 (5.3%)	0 (0.0%)	4 (10.5%)
	Between 16 and 30 min	5 (13.2%)	22 (57.9%)	4 (10.5%)	6 (15.8%)	17 (44.7%)
	More than 30 min	0 (0.0%)	4 (10.5%)	0 (0.0%)	2 (5.3%)	2 (5.3%)

Abbreviations min/day = Minute per day, h/day = Hour per day, nº/day = Number per day.

The frequency range of dog walks showed statistically significant differences when observed by the employment status of owners (*p* = 0.029). The remaining variables of the owner-reported PA of dogs did not show significant differences in relation to employment and education level. The associations between the remaining demographic variables of the owners and the PA performed by the dogs are presented in Table 5.

**Table 5:** Association between demographic variables of the owners and physical activity (PA) of the dogs

Variables	Owner-Reported PA of Dogs			Dogs’ Accelerometry					
	Duration of dog walking (min/day range)	Time dogs spend alone (h/day range)	Freq of walks (nº/day range)	Sedentary time (min/day)	METs (kcal/kg/h)	Step Counts (counts/day)	MPA (min/ day)	VPA (min/day)	MVPA (min/ day)
**Spearman Correlation (Rho)**									
**Owners**									
Age	0.279	-0.104	-0.090	0.069	0.046	-0.044	0.164	0.128	0.165
BMI	-0.006	0.217	-0.013	0.294	-0.156	-0.146	-0.108	-0.061	-0.11
Household composition	-0.415**	0.047	0.222	-0.237	-0.104	-0.184	-0.127	0.036	-0.110

Additionally, the education levels of owners demonstrated a moderately significant correlation with the level of importance attributed to their dog’s health (rho = 0.385, *p* = 0.01).

Owners who reported higher SRH scores also show a moderately significant correlation with their dog’s weight (rho = 0.390, *p* = 0.02). There was also a moderately significant relationship between the importance of the dog’s health for owners and the duration of dog walking (rho = -0.320, *p* = 0.05).

Moderately significant associations were identified between LPA by DO and VPA by dogs (rho = 0.445, *p* = 0.01). The practice of MVPA by DO showed a moderate association with the size categories of the dogs (rho = 0.355, *p* = 0.03).

Dogs with a higher BMI shower significant correlation with the frequency at which their owners took them for walks (rho = -0.561, *p* = 0.00). Conversely, the sedentary activity time of the dogs had a moderately significant negative correlation with the frequency at which their owners took them for walks (rho = -0.411, *p* = 0.01).

### Lifestyle

In relation to lifestyle, the results suggest that DO with a healthier lifestyle, as measured by the FANTASTICO, exhibited moderately significant correlation with the SRH scores of owners (rho = 0.391, *p* = 0.02). The importance of dog’s health, as perceived by owners, is moderately associated with the FANTASTICO scale (rho = 0.398, *p* = 0.01), highlighting the moderate significant correlation subscale Family and Friends (rho = 0.398, *p* = 0.01), and the highly significant correlation with Other Behaviors (rho = 0.671, *p* = 0.00). For more detailed information on the correlations between the FANTASTICO subscales and variables related to PA Accelerometer data in pets, please see Table 6.

**Table 6:** The association between physical activity (PA) of dogs and the FANTASTICO lifestyle subscales of dog owners

Variables (Rho)	F	A	N	T	A	S	T	I	C	O	
**Dog accelerometer data**	0.246	0.157	0.007	0.122	-0.212	0.323*	0.030	0.317	0.194	0.215	
**METs (kcal/kg/h)**	0.210	0.254	0.107	-0.122	-0.364*	0.375*	0.184	0.326*	0.083	0.079	
**Steps (Counts/day)**	0.180	0.176	-0.007	-0.068	-0.288	0.390*	0.178	0.331*	0.115	0.076	
**MPA (min/day)**	0.076	0.005	-0.112	0.037	-0.082	0.141	0.091	0.119	0.019	0.196	
**VPA (min/day)**	0.162	0.178	-0.020	-0.083	-0.284	0.393*	0.167	0.307	0.094	0.059	
**MVPA (min/day)**	0.246	0.157	0.007	0.122	-0.212	0.323*	0.030	0.317	0.194	0.215	

Abbreviations kcal/kg/h = Kilocalorie per kilogram per hour, METs = Number of metabolic equivalents, min/day = Minute per day, counts/day = counts per day, MPA = Moderate PA, MVPA = Moderate-to-vigorous PA, VPA = Vigorous PA. * *p* ≤ 0.05, F - Family and Friends; A - Associativism/physical Activity; N - Nutrition, T- Tobacco, A- Alcohol and other drugs; S - Sleep/Stress; T - Type Personality/Work; I - Introspection; C - Health and Sexual Behaviors; and O - Other Behaviors.

Considering the subscales of the FANTASTICO Scale, owners who achieved higher scores in the Personality/Work subscale and exhibited better Other Behaviors subscales showed a moderately significant correlation with lower BMI values of their dogs (rho = -0.345, *p* = 0.04, and rho = -0.323, *p* = 0.05, respectively). The values obtained in the Family and Friends subscale and in the Introspection subscale also showed a moderately significant negative correlation with the dog’s age (rho = -0.483, *p* = 0.00, and rho = -0.423, *p* = 0.01, respectively). A moderately significant association was found between the Smoking Habits subscale and longer periods of dogs staying at home alone (rho = 0.395, *p* = 0.02), as well as the PA of dogs measured by accelerometry, particularly in relation to their daily step counts (rho = 0.353, *p* = 0.03).

### Attachment

Regarding attachment to the dog, the Proximity LAPS subscale exhibited significant moderate correlations with female gender (rho = 0.446, *p* = 0.01), owners who reported walking more frequently with their dogs (rho = 0.358, *p* = 0.03), and the Associativism/Physical Activity subscale of the FANTASTICO (rho = -0.433, *p* = 0.01).

DO with higher scores on the Importance subscale demonstrated a significant moderate correlation with the Associativism/Physical Activity subscale of the FANTASTICO (rho = -0.414, *p* = 0.01). Additionally, the Importance subscale showed a large significant correlation with the frequency of walking with their dogs (rho = -0.785, *p* = 0.02).

The values obtained in the Bond Subscale demonstrated a significant moderate correlation with SRH scores (rho = 0.337, *p* = 0.04), more frequent walks with the dog (rho = 0.343, *p* = 0.04), and lower consumption of Alcohol and Other Drugs (FANTASTICO subscale) (rho = 0.372, *p* = 0.02).

Overall, individuals who were more attached to their dogs showed a moderate correlation with the frequency of walks with the dog (rho = 0.392, *p* = 0.01), the Associativism/Physical Activity subscale (rho = -0.372, *p* = 0.02), and the Nutrition subscale (FANTASTICO) (rho = 0.356, *p* = 0.03).

## Discussion

This exploratory study sought to examine whether the attachment between dog owners (DO) and their dogs influenced total physical activity (PA), as measured by accelerometry. Additionally, we examined the relationship between socio-demographic variables, lifestyle factors, the SRH of the DO and the health care of their dogs.

This study was conducted in a real-life context, which provides a unique approach to capturing the actual levels of PA between DO and dogs. Additionally, it adopts a One Health approach, considering the benefits for both DO and dogs. Moreover, to the best of our knowledge, this is the first study conducted in Portugal to provide an objective assessment of PA in both dogs and DO, simultaneously. Furthermore, it includes a comprehensive analysis of various measures (sociodemographic and health-related) that exhibit different associations among the variables studied. Additionally, an analysis was conducted on the human-animal bond, yielding valuable insights into its influence on levels of PA for both DO and dogs. Our findings provide insights into how DO’s socio-demographic characteristics influence their dogs. The results revealed that the majority of DO have a healthy profile, characterized by a normal BMI, good health perception, and a very good lifestyle. It is worth noting the high educational level of the sample, which likely contributed to the development and adoption of healthy behaviors, as reported in other studies [74, 75]. Interestingly, our results provide evidence that this profile of health-conscious DO is associated with the health care of their dogs. Specifically, factors such as DO’s age, education, and occupation were identified as factors associated with control over their dog’s weight. DO working in the tertiary sector were associated with higher levels of PA for their dogs. Furthermore, a correlation was observed between higher levels of DO education and employment, which were linked to concerns about their dogs’ caloric expenditure and levels of PA. This association aligns with the findings of Silva et al. [48] and can be attributed to increased knowledge and awareness of pet’s health, which is necessary for a more responsible approach to pet adoption [76].

In this study, a particularly intriguing relationship between employed DO and the PA of their dogs, as measured by accelerometry, was identified. This relationship may be attributed to the structured schedules of employed owners, which could lead to more consistent exercise routines with their dogs. Indeed, a significant relationship was observed between the frequency of dog walks (three times daily) and employment status. On the other hand, considering unemployed owners, who have a reduction in full-time work obligations, it would be expected that the PA levels of their dogs would be higher in this group. Other factors may have influenced this relationship, however due to the nature of variable, a multivariate approach was not supported. These analyses should be considers in future studies.

When examining the various parameters of the lifestyle scale, it is noticeable that DO who reported greater family support, fewer smoking habits, heightened awareness of their own health and sexual behaviors, or engaged in other positive behaviors tended to place greater importance on their dog’s health. These associations emphasize the interconnectedness between human and dog well-being. Adopting healthy habits allows DO to positively influence their dogs’ health and well-being, reinforcing the notion of a symbiotic relationship between human and pet’s health. Conversely, this study confirmed previous findings that DO with negative health habits, such as smoking, show less concern for their dog’s well-being and engage in less PA with their dogs [77].

Additionally, our study found that DO from larger households tended to have shorter walks. This relationship could possibly be related to the fact that in larger households, multiple family members may share the responsibility of walking the dog, resulting in shorter walks that are more manageable for the entire family. Furthermore, the presence of children or older family members in larger households may limit the ability to participate in longer walks, thereby influencing the duration of the walks undertaken. Future studies should consider this variable during sample recruitment.

The dogs in our study consisted of a wide range of ages, reflecting a diverse representation of dogs across different life stages. Interestingly, we found a negative association between the importance of Family and Friends subscale on the FANTASTICO scale and the dogs’ age. This sub-scale includes items related to having someone to talk to about the DO’s problems and feeling emotionally supported. Possibly, to DO who do not have other family members or friends to confide in, their animal companion often serves as a source of companionship and emotional support. Moreover, it is expected that the role of a pet as a confidant increases as the years pass. According to Bowen (2021) a dog that is present in a household, should be regarded as an important resource for social support. Therefore, our results suggest that owners who have friends and family members to talk about their lives, do not share their problems with their pets. On the other hand, those that have difficulty sharing their problems and concerns use their old friend (dog) as a confidant.

Regarding dog breeds, a substantial portion of the sample in our study consisted of crossbreeds. While many studies tend to focus on dogs of specific breeds [78, 79], which can be valuable for analysis purposes, it may not fully capture the diversity of dogs that constitute a significant portion of adopted dogs by Portuguese families. Additionally, our results indicate that larger dog breeds were associated with higher levels of MPVA by the DO.

Considering BCS [56–58], our findings revealed that 55.3% of the dogs were overweight. Similar to humans, obesity in dogs is associated with lower levels of PA [12], which aligns with our findings of a significant association between higher BMI in dogs (BCS scores) and a lower frequency at which their owners take them for walks. Additionally, we also found that dogs with higher body weight were associated with those that spend more time alone at home. Numerous studies have reported a concerning increase in the prevalence of overweight and obese dogs globally, which has detrimental consequences for their health, lifespan, and quality of life [58, 80–82]. Furthermore, there is evidence of an association between human obesity and canine obesity, indicating shared environmental and lifestyle factors contributing to both conditions [83].

On the other hand, research has demonstrated that DO who engage in regular walking with their dogs are less likely to be obese compared to both non-dog owners and DO who do not walk with their dogs [84]. This reinforces the notion that the lifestyle choices of DO, including their exercise habits for their pets, play a significant role in determining the body weight and overall health of dogs [80, 85].

Additionally, it was observed that DO who placed greater importance on their pet’s health were more likely to engage in regular walks with their pets, as also observed by Rohlf et al. (2012) [32]. This suggests that DO recognize the health benefits associated with exercise and seek to extend them to their dogs.

In terms of measuring PA levels using accelerometry, one of the criteria for this study was to assess the PA of both DO and dogs in their daily routine. This approach was chosen because it provides a more realistic representation of the pets’ activity levels within each family system. It takes into account that a dog’s PA can vary from day to day and may be influenced by the activities of their DO [86]. This could contrast with a laboratory environment where dogs’ routines are often more standardized. However, one limitation of approach adopted in this study is that these activities are more diverse and uncontrolled, which can result in higher variability in the activity counts.

This study revealed that a high percentage of DO were found to comply with WHO recommendations for MVPA [63]. This indicates that the majority of DO engage in a mean of 150 to 300 min per week of MVPA, which is considered beneficial for health. However, while DO individually meet the MVPA recommendations, the association with dogs’ PA was only found between DO’s LPA and dog’s MVPA. This suggests that DO typically walk with their dogs but do not engage in running activities, which is consistent with previous studies [87–90]. On the other hand, a study by Richards et al. [91] found that young adult DO spend the majority of their time walking their dogs at MVPA intensity levels. This may suggest that the DO’s walking pattern could depend on their age.

Our results, despite being based on a small sample, are significant in promoting PA through dog walking [92]. In Portugal, approximately 38% of households own a dog [16, 93]. Dog walking can contribute to achieving sufficient levels of LPA among DO, potentially in resulting health benefits [63]. DO tend to engage in light to moderate PA more frequently than those without dogs [94]. Moreover, combining of LPA and MVPA has been shown to provide health benefits [95]. In addition, research studies have consistently highlighted the numerous advantages of pet ownership in fostering PA across a range of age groups [23, 88, 96–98]. In 2013, a meta-analysis of 17 studies demonstrated that DO engage in more walking and overall PA compared to non-dog owners [18]. Additionally, a study by Soares et al. [20] revealed that 63.9% of DO reported walking their dogs, and DO were more than 2.5 times more likely to achieve PA guidelines. Recently, Martins et al. [23] confirmed that the presence of pets had a positive effect on the PA levels of DO compared to non-pet owners and that pet owners engaged in PA more frequently than non-pet owners.

According to Yam et al. [39], the PA performed by dogs can range from light to moderate intensity. This corresponds to a slow to moderate translocation of the trunk while the dog is on a leash. On the other hand, vigorous PA involves a rapid translocation of the trunk while the dog is running off a leash, typically outdoors. Therefore, taking the dog for a walk outside allows them to achieve exercise intensity levels that can help prevent canine obesity. However, whether or not dogs can walk without a leash may depend on their behavioral characteristics.

Research has shown that the bond between owners and dogs contributes to the amount of time spent walking by the owners [99]. This study found that the more emotionally attached an owner is to their dog, the more frequently they go on walks together. These findings highlight the role of pets as a source of motivation and social support for engaging in PA [23, 90]. We believe that the emotional bond between DO and their dogs can serve as a powerful motivator for increasing PA levels. Having a beloved pet encourages owners to be more active, and committed to activities like walking and exercising with their dogs. Additionally, stronger emotional bonds often lead owners to seek new experiences and activities to share with their four-legged companions. This includes participating in activities that owners believe their dogs will enjoy. These emotional connections tend to grow through shared experiences, so it is not surprising that individuals who lead active lives are more likely to engage in a variety of activities with their dogs.

Conversely, it is plausible that individuals with preexisting active lifestyles are drawn to the prospect of dog ownership. This inclination may result in higher levels of PA. We propose that future research endeavors to investigate the causal relationship between the strength of the human-dog bond and the upsurge in owner PA. The aim is to determine whether the intensity of the bond justifiably accounts for the higher PA levels, or if the owner’s active lifestyle predisposes them to increased PA.

Women demonstrated a stronger bond with their dogs, which can be explained by the fact that women exhibited greater emotional closeness with their dogs compared to men. This finding aligns with previous studies [33, 87, 88]. Dog walks provide opportunities for increased interaction and bonding,

leading to various developmental benefits such as improved self-esteem, self-regulation, and empathy [100]. Additionally, in particular situations of isolation, such as during the COVID-19 pandemic, dog walking has been found to directly impact attachment and indirectly influence the perceived loneliness of DO [101]. Therefore, increasing PA through the attachment between humans and dogs can be a valuable approach to benefit both owners and their pets, aligning with the goals of the One Health approach [11].

Despite the promising results, it is important to acknowledge several limitations in this study. Firstly, the sample size, while sufficient for exploratory purposes, was relatively small and may not comprehensively represent the diversity of DO within the broader population. Additionally, although many relationships exhibited statistically significant correlations, these correlations were often modest, warranting caution in interpretation. Future research endeavors could potentially benefit from employing a multivariate approach, thereby providing a more comprehensive understanding of the complex interplay of variables within our study domain. Furthermore, lifestyle and attachment data relied on self-reports from owners, which can introduce response biases. The potential for selection bias is another consideration, as voluntary participation by participants may indicate some predisposition toward an interest in the human-animal bond or their dog’s health.

On the other hand, it is noteworthy that this study was conducted during the spring season when favorable temperatures and reduced precipitation encourage outdoor walking. However, it is worth mentioning that previous studies have reported that DO are more inclined to engage in outdoor recreational walking, even during winter compared to non-dog owners [102].

While these findings provide valuable insights, further investigation in larger and more diverse populations is necessary to replicate the results. Nevertheless, the outcomes of this small-scale study suggest that dog walking can be a valuable approach to achieving greater health benefits, deserving recognition and promotion. To enhance future research in this field, considerations should include larger and more diverse sample sizes, longitudinal study designs, inclusion of control groups, and the incorporation of objective health metrics to address these limitations and bolster the robustness of our understanding of the relationships explored in this study.

## Conclusion

In conclusion, the findings of this research emphasize that individuals who adopt healthier habits tend to perceive themselves as healthier and show increased concern for their pets’ health. Moreover, there is evidence supporting the emotional bond between owners and dogs, as well as higher levels of PA. This positive association between dog ownership and health has important implications for community health, as it can potentially contribute to the reduction of healthcare costs at a broader level. In this regard, our findings add nuances to current research by recognizing that the benefits of having a dog should be seen as a spectrum of multiple mutual influences between the dog and the owner, ultimately resulting in a healthy lifestyle for both and a reduction in non-communicable diseases.

### Electronic supplementary material

Below is the link to the electronic supplementary material.

Supplementary Material 1

Supplementary Material 2

## Data Availability

The datasets generated and/or analyzed during this study will be available upon reasonable request from the corresponding author.

## Abbreviations

BCS: Body Condition Score
BMI: Body mass index
DO: Dog owner
kcal/kg/h: Kilocalorie per kilogram per hour
LAPS: Lexington attachment to pet’s scale
LPA: Light PA
METs: Number of metabolic equivalents
min/day: Minute per day
MPA: Moderate physical activity
MVPA: Moderate-to-vigorous physical activity
nº/day: Number per day
PA: Physical activity
SD: Standard deviation
SRH: Self-rated health
VPA: Vigorous physical activity
WHO: World Health Organization
