# Supplementary material for: Walk or be walked by the dog? The attachment role
Source: BMC Public Health. 2024 Mar 4;24:684. doi: 10.1186/s12889-024-18037-4 (PMC10913448; doi:10.1186/s12889-024-18037-4)

**Supplementary Figure 2**

Counts recorded by accelerometry for each minute of a 24-hour day for a dog owner (DO) and respective dog during the same period (an example).


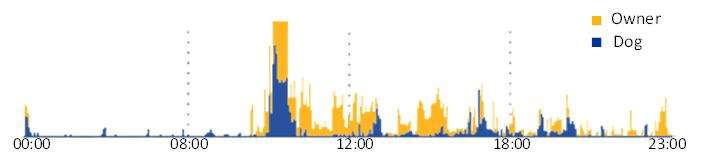

Supplement: Supplementary file 1 — Supplementary Material 1 [file 12889_2024_18037_MOESM1_ESM.docx]
