# Supplementary material for: Walk or be walked by the dog? The attachment role
Source: BMC Public Health. 2024 Mar 4;24:684. doi: 10.1186/s12889-024-18037-4 (PMC10913448; doi:10.1186/s12889-024-18037-4)
Supplement: Supplementary file 2 — Supplementary Material 2 [file 12889_2024_18037_MOESM2_ESM.docx]

**Supplementary table 1**

The Portuguese version of The Lexington Attachment to Pets Scale (LAPS) (Midanda, 2011).

| **1 – Discordo plenamente** | **2 – Discordo em parte** | **3 – Não sei ou recuso responder** | **4 – Concordo em parte** | **5 – Concordo plenamente** |
| --- | --- | --- | --- | --- |

| Frase | Grau | | | | |
| --- | --- | --- | --- | --- | --- |
| 1. O meu animal de estimação significa mais para mim do que qualquer um dos meus amigos. | 1 | 2 | 3 | 4 | 5 |
| 2. Muitas vezes eu confidencio com o meu animal de estimação. | 1 | 2 | 3 | 4 | 5 |
| 3. Eu acredito que os animais de estimação deveriam ter os mesmos direitos e privilégios que os membros da família. | 1 | 2 | 3 | 4 | 5 |
| 4. Eu acredito que o meu animal de estimação é o meu melhor amigo. | 1 | 2 | 3 | 4 | 5 |
| 5. Muitas vezes, os meus sentimentos acerca das pessoas são influenciados pela forma como reagem ao meu animal de estimação. | 1 | 2 | 3 | 4 | 5 |
| 6. Eu adoro o meu animal de estimação porque ele/ela é mais leal do que a maioria das pessoas que eu conheço. | 1 | 2 | 3 | 4 | 5 |
| 7. Eu gosto de mostrar a outras pessoas fotografias do meu animal de estimação. | 1 | 2 | 3 | 4 | 5 |
| 8. Eu penso que o meu animal de estimação é apenas um animal de estimação. | 1 | 2 | 3 | 4 | 5 |
| 9. Eu adoro o meu animal de estimação porque ele nunca me julga. | 1 | 2 | 3 | 4 | 5 |
| 10. O meu animal sabe quando me sinto mal. | 1 | 2 | 3 | 4 | 5 |
| 11. Eu falo muitas vezes com outras pessoas acerca do meu animal de estimação. | 1 | 2 | 3 | 4 | 5 |
| 12. O meu animal de estimação compreende-me. | 1 | 2 | 3 | 4 | 5 |

| Frase | Grau | | | | |
| --- | --- | --- | --- | --- | --- |
| 13. Eu acredito que gostar dos meus animais de estimação ajuda a manter-me saudável. | 1 | 2 | 3 | 4 | 5 |
| 14. Os animais de estimação merecem tanto respeito como os  humanos. | 1 | 2 | 3 | 4 | 5 |
| 15. Eu e o meu animal de estimação temos uma relação muito próxima. | 1 | 2 | 3 | 4 | 5 |
| 16. Eu faria quase tudo para cuidar do meu animal de estimação. | 1 | 2 | 3 | 4 | 5 |
| 17. Eu brinco muitas vezes com o meu animal de estimação. | 1 | 2 | 3 | 4 | 5 |
| 18. Eu considero que o meu animal de estimação é uma grande companhia. | 1 | 2 | 3 | 4 | 5 |
| 19. O meu animal de estimação faz-me sentir feliz. | 1 | 2 | 3 | 4 | 5 |
| 20. Eu sinto que o meu animal de estimação faz parte da minha família. | 1 | 2 | 3 | 4 | 5 |
| 21. Eu não sou muito próximo/a do meu animal de estimação. | 1 | 2 | 3 | 4 | 5 |
| 22. Ser dono do meu animal de estimação faz parte da minha felicidade. | 1 | 2 | 3 | 4 | 5 |
| 23. Eu considero que o meu animal de estimação é um amigo. | 1 | 2 | 3 | 4 | 5 |
